# Investigation of Olea ferruginea Roylebark extracts for potential in vitroantidiabetic and anticancer effects

**DOI:** 10.3906/kim-2006-51

**Published:** 2021-02-17

**Authors:** Samra LIAQAT, Muhammad ISLAM, Hamid SAEED, Mehwish IQTEDAR, Azra MEHMOOD

**Affiliations:** 1 University College of Pharmacy, University of the Punjab, Lahore Pakistan; 2 Department of Biotechnology, Lahore College for Women University, Lahore Pakistan; 3 Center for Excellence in Molecular Biology, University of the Punjab, Lahore Pakistan

**Keywords:** *Olea ferruginea* R, cytotoxicity, MTT, Akt, BAD, antidiabetic

## Abstract

This study was conducted to investigate the physicochemical, phytochemical, in vitro antidiabetic and anticancer potential of *Olea ferruginea* R bark. After extraction using Soxhlet, in vitro antidiabetic and cytotoxic activity on human hepatocellular carcinoma (HepG2) cells was assessed by nonenzymatic glycosylation of hemoglobin assay, alpha-amylase inhibition assay, glucose uptake by yeast cells, and 3-[4,5-dimethylthiazol-2-yl]-2,5 diphenyl tetrazolium bromide assay, respectively, and gene expression via real-time polymerase chain reaction. Primary and secondary metabolites were present in the extractants; polyphenols (35.61 ± 0.03) and flavonoids (64.33 ± 0.35 **)** in the chloroform; and polysaccharides in the ethanol (268.75 ± 0.34), and glycosaponins (78.01 ± 0.07) in the methanol. The chloroform extract exhibited maximum antidiabetic potential, showing inhibition of nonenzymatic glycosylation of hemoglobin (65%), and alpha-amylase inhibition (32%) with maximum percent glucose uptake by the ethanol extract (78%). Only the ethanol extract had dose-dependent cytotoxic potential against the HepG2 cells. After 24-h exposure to the ethanol-extract, the expression of protein kinase B (Akt) remained unchanged, while the expression of B-cell lymphoma 2 (BCL2) and BCL2 associated X (BAX) changed significantly. After 48-h exposure, the expression of Akt decreased significantly, while that of BCL2 and BAX increased significantly. *Olea ferruginea* R bark possessed in vitro antidiabetic potential and anticancer/cytotoxic effects, attributable to the decline in the prosurvival signals of the Akt signaling pathway.

## 1. Introduction

Medicinal plants have been utilized to treat various ailments all over the world, although specific to every region [1]. Regarded as a natural treasurer, these sources are plentiful and can be utilized in galenical products in a safe, standardized, and effective manner with subsequent use in primary health care facilities [2]. History has revealed that humans have been benefiting from these herbal consecrations for almost 60,000 years and they can be consumed either directly or in the form of the active constituent isolated from them [3]. As of today, an estimated 50,000 medicinal plant species have afforded health benefits to approximately 80% of the world’s population [4]. Pakistan, with climatic variations and rich soil, offers the finest conditions for the growth of flora across the country. It is estimated that about 12% of the flora in Pakistan is used for medicinal purposes, while some of them are exported [5]. Almost 6000 plant species have been discovered in Pakistan, of which 1000 are used for medicinal purpose [6]. It has also been estimated that 70% of the total species are uniregional and about 30% are bi- or pluriregional. The country has four phytogeographical regions, comprising Irano-Turanian (45% of the species), Sino-Himalayan (10%), Saharo-Sindian (9.5%), and the Indian element (6%) [7].

*Olea ferruginea* R, locally known as Indian olive or Kahoo [8], a well-known medicinally important plant, is widely distributed in different areas of Pakistan, such as Swat valley, Salt Range, Soon valley, the western hills of Balochistan, lower hills of Azad Kashmir, Waziristan, Dir, Chitral, and Murree Hills [9]. The genus *Olea* includes around 30 to 40 species that are distributed in Oceania, Asia, Africa, and the Mediterranean Basin [10]. Numerous reports have suggested that different parts of *Olea ferruginea* R have many traditional uses, such as the leaves, which are used to treat bleeding gums, gonorrhea, whooping cough, skin problems, skeleton-muscular problems, and tooth ache, while its bark is used to treat fever [11,12] and the dried fruit has an antidiabetic effect [13]. Additionally, the olive oil extracted from its fruit and seeds reduces swelling and pain related to rheumatoid arthritis [14]. The leaves of *Olea ferruginea* R possess glucose lowering and cholesterol lowering potential [15]. Moreover, *Olea ferruginea* R leaves have demonstrated in vitro an alkaline phosphatase inhibitory effect on human cervical cancer cells (HeLa cancer cell-lines) with cytotoxicity, confirmed via sulforhodamine B (SRB) assay [16].

 Oleanolic acid, a biologically active compound isolated from the chloroform extract of *Olea ferruginea* R, has been shown to exhibit antitumor, antimicrobial, hepatoprotective, and antiallergic potential [17], while neuroprotective effects were observed in a focal brain hypoxia rat model [18]. Moreover, the fruit of *Olea ferruginea* R were reported to possess antioxidant [19] and hepatoprotective activity against fluoride-induced toxicity in mice [20]. More recently, the bark and leaves of *Olea ferruginea* R have shown to possess antimicrobial and antioxidant potential [21]. The isolated constituents from *Olea ferruginea* R stem, ferruginan and cycloolivil, have been shown to possess antibacterial and antileishmaninal activity [22].

According to an estimate, 150,000 cases of cancer arise in Pakistan annually, of which 60%–80% individuals die as a result [23]. Likewise, diabetes mellitus is yet another highly prevalent disease in Pakistan [24]. A lack of literature evidence exists regarding the antidiabetic and anticancer potential of *Olea ferruginea* R bark extract. Thus, the present study aimed at investigating *Olea ferruginea* R bark extracts for their in vitro antidiabetic activity using nonenzymatic glycosylation of hemoglobin assay, alpha-amylase inhibition assay, glucose uptake by yeast cells and in vitrocytotoxic effect using the human liver carcinoma cell line, HepG2.

## 2. Materials and methods

### 2.1. Collection of the plant material

On 15 July 2017, the bark of *Olea ferruginea* R was collected from Soon valley, District Khushab, Punjab, Pakistan, and was recognized and authenticated by the Department of Botany, Government College University, Lahore, Pakistan (GC. Herb. Bot. 3499), under collector number: 0307-5558886. The bark was cleaned and sun dried for a week, and then pulverized after drying and stored for later use.

### 2.2. Chemicals and reagents

Chemicals and solvents of analytical grade used in the experiments are as follows: sulfuric acid, n-Hexane (British Drug Houses, London, England, UK), methanol, chloroform, ethanol, potassium acetate, hydrochloric acid, nitric acid, cooper sulphate, sodium carbonate, bovine serum albumin, aluminum nitrate, sodium hydroxide, acetone (Merck, Darmstadt, Germany), quercetin (Sigma Life Science, Darmstadt, Germany), gallic acid (Sinochem, Beijing, China), triton X (Unichem Chemicals, Dublin, Ireland, UK), Folin-Ciocalteu reagent (Unichem Chemicals), anthrone reagent (Sigma Life Sciences), sodium chloride (Merk), sodium phosphate monobasic (Sigma Aldrich, St. Louis, MO, USA), Di sodium hydrogen phosphate (Riedel-de Haen, Seelze, Germany), alpha-amylase (EYER Chemical Reagent Ltd., Shanghai, China), Starch (China), acarbose (Glucobay 100 mg; Bayer Pharma, Leverkusen, Germany), 3,5,dinitro salicylic acid (Central Drug House, New Delhi, India), potassium sodium tartarate (British Drug Houses), hemoglobin, glucose (Daejung Co., Ltd., Busan, Korea), gentamycin (Ray Pharma, Karachi, Pakistan), tocopherol (Merck), metronidazole (Sanofi Aventis, Karachi, Pakistan), yeast (Halal, Food Empire Pvt, Ltd.), distilled water, dimethyl sulfoxide (DMSO) (Daejung Co., Ltd.), fetal bovine serum (FBS) (HyClone, Cramlington, Northumberland, UK), penicillin-streptomycin solution (HyClone), phosphate buffer saline (Oxoid, England), Dulbecco’s modified eagle medium (DMEM) (HyClone), (3-[4,5-dimethylthiazol-2-yl]-2,5 diphenyl tetrazolium bromide (MTT) reagent (bioPlus Research Chemicals, Dublin, Ireland), trypsin (HyClone), isopropanol (British Drug Houses), trizol (BioShop Canada Inc. Burlington, Ontario, Canada; Catalogue #RT 111, RL 311), Hyperscript first strand synthesis kit (Catalogue #601-005), syber green (Bioshop), nuclease-free water (Bioshop), and purified water (Caisson, North Logan, UT, USA).

### 2.3. Physicochemical analysis of the powdered bark

In accordance with United States Pharmacopoeia procedures as described in [25] the moisture content, total ash, water soluble ash, acid insoluble ash, sulphated ash, and water and alcohol soluble extractive values were estimated.

### 2.4. Determination of the mineral contents

The mineral contents of 1 g of powdered bark of *Olea ferruginea* R were determined according to the method described by Ayeni et al. [26].

### 2.5. Extraction of the plant powdered bark

#### 2.5.1. Hot extraction:

Hot extraction was performed on the powdered material of *Olea ferruginea* R using a Soxhlet apparatus. The solvents were used in the increasing order of polarity, which comprised n-Hexane (B.P 68 °C), chloroform (B.P 61.2 °C), and methanol (B.P 65 °C). For this procedure, 60 g of powdered material was packed into the filter paper and then it was placed in the form of a thimble in the Soxhlet apparatus. Next, 1.5 L of n-hexane was added and extraction was performed until the siphon was transparent. Following that, the extract was collected in glass vials. This whole procedure was then repeated on the same material using chloroform and methanol as solvents.

#### 2.5.2. Cold extraction

Ethanol and aqueous extracts were obtained by the maceration technique. First, 25 g of powdered material was allowed to macerate with 250 mL of ethanol in a beaker, which was then stirred over a magnetic plate for 24 h. The beaker was covered with aluminum foil to avoid solvent loss. After 24 h, the filtrate was collected and residue was again extracted with fresh solvent, and after 1 h, the mixture was filtered. The same procedure was repeated again on the same material using fresh solvent. The 3 ethanol filtrates were collected in the same beaker. The aqueous extract was collected using distilled water in place of the ethanol in same quantity using the same procedure.

#### 2.5.3. Collection of the extracts

All of the extracts were dried in a rotary evaporator, except for the aqueous extract, which was freeze dried. All of the extracts were collected in proper labelled, tarred, sample vials and these vials were kept in an oven at 40 °C until all of the solvent had evaporated.

### 2.6. Determination of primary and secondary metabolites

The bark extracts were subjected to phytochemical screening to determine the primary and secondary metabolites. The primary metabolites included the total protein, lipid, and carbohydrate contents, which were evaluated according to a protocol described previously [27–29], while the secondary metabolites consisted of the total polyphenol, flavonoid, polysaccharide, and glycosaponins contents, and were measured in accordance with the standard official procedures [30–32].

### 2.7. In vitro antidiabetic activity

#### 2.7.1. Nonenzymatic glycosylation of hemoglobin assay

The nonenzymatic glycosylation of hemoglobin assay was performed, as reported previously [33], with slight modifications. The *Olea ferruginea* R bark extracts were prepared in 0.01 M of phosphate buffer (pH 7.4) at a concentration of 1 mg/mL. Precisely, 1 mL of 0.06% hemoglobin solution, 5 µL of 0.02% gentamycin, 1 mL sample solution, and 1 mL of 2% glucose solution was mixed. All of the reagent solutions were also prepared in the buffer. The mixture was incubated for 72 h at 37 °C in a dark environment. Thereafter, the degree of glycosylation was measured at 443 nm [34] using UV-Vis spectrophotometry. Tocopherol was used as a standard drug. Determination of the percentage inhibition was calculated using the formula in Eq. (1).

Percentage inhibition=Absorbance of control - Absorbance of sampleAbsorbance of controlx100

#### 2.7.2. Alpha-amylase inhibition assay 

The alpha-amylase inhibition assay was performed as described previously [35], with some modifications. Briefly, 1 mL of alpha-amylase solution (1% w/v in sodium phosphate buffer, pH 6.9) and 1 mL of sample solution (1 mg/mL in sodium phosphate buffer, pH 6.9) were taken and incubated at 37 °C for 5–15 min. Thereafter, 1 mL of potato starch solution (1% w/v in sodium phosphate buffer, pH: 6.9) was added and incubated for 15 min followed by the addition of 1 mL 3,5-dinitro salicylic acid color reagent. The whole mixture was kept in water bath that was maintained at 85 °C for 10 min. Later, once it had cooled at room temperature, the absorbance was measured at 540 nm using UV-Vis spectrophotometry. Acarbose was used as a standard drug. The extracts were tested for their antidiabetic potential and the percentage inhibition was calculated using the formula in Eq. (1).

#### 2.7.3. Glucose uptake by yeast cells 

Glucose uptake by the *Olea ferruginea* R bark extracts was evaluated using yeast cells assay as described previously [36], with some modifications. Distilled water was used to prepare yeast suspension (10% v/v), extract solutions (1 mg/mL), and 10mM glucose solution. Next, 1 mL of sample solution and 1 mL of 10 mM glucose solution were mixed and incubated at 37 °C for 10 min. The reaction was started by adding 100 µL of yeast suspension. After which, the whole mixture was vortexed for 1 min and incubated at 37 °C for 1 h. After centrifugation at 3000 rpm for 15 min, glucose was quantified in the supernatant by measuring the absorbance at 620 nm using UV-Vis spectrophotometry. Metronidazole was used as standard in this assay. Determination of the percentage increase of glucose uptake by the yeast cells was calculated using the formula in Eq. (2). 

Percentage increase in the glucose uptake =Absorbance of control - Absorbance of sampleAbsorbance of controlx100

### 2.8. Cell culture for cytotoxic assay

The HepG2 cell line was very kindly provided by Dr. Azra Mehmood, of the Center of Excellence in Molecular Biology, Punjab University, Lahore, Pakistan. For the HepG2 cell culture, DMEM was supplemented with 10 % FBS and 1% penicillin-streptomycin and maintained in the incubator at 37 °C, 5% CO_2_, and 95% relative humidity. The cells were expanded in a T-75 flask, and for the experiments, at 80%–90% confluency, the cells were lifted with 3 mL of trypsin/EDTA for 5–10 min, with gentle shaking, followed by media flushing to make a single cell suspension. The lifted cells were then transferred into a 15-mL falcon tube for centrifugation at 2000 rpm for 10 min to pellet the cells. The pelleted cells were then resuspended in fresh media (5–10 mL) and counted via a Neubauer chamber in the 4 corner squares of the 16 squares, and the average was calculated. For counting, the cells in the middle of the square and those touching the middle lines on the top and left border were included, while cells touching the middle line on the bottom and right were excluded. The total number of cells in a volume were counted by multiplying the average number of cells counted in the 4 corner squares with the dilution and conversion factor (104).

#### 2.8.1. MTT assay 

MTT assay was performed as described previously [37] to assess the cytotoxic capacity of the powdered *Olea ferruginea* R bark extracts. First, 50 and 100 µg/mL of the bark extract concentrations were used to check the cytotoxicity effects on the HepG2 cells. Briefly, 200 µL (total 2000 cells) of the cell suspension was seeded into each well of a 96-well plate. Next, the plate was kept in an incubator for 24 h at 37 °C, 95% relative humidity, and 5% CO_2_. After 24 h, the plate was examined for expansion of the HepG2 cells using an inverted microscope. When almost 70% area of each well was covered with the cells, then the media was removed from each well carefully, without disturbing the cells. These cells were then exposed to 200 µL of both concentrations (50 µg/mL and 100 µg/mL) of each extract and incubated at 37 °C, 5% CO_2_ and 95% relative humidity for 24 h. Different concentrations were prepared in triplicate to minimize the error. The following day, on completion of the 24 h, 20 µL of MTT reagent was added and incubated for 4 h under the same conditions described above. The concentration of MTT reagent was 5 mg/mL using DMSO as a solvent. This was followed by the removal of 80% of the contents from all of the wells. Next, 150 µL of DMSO was added to each well for the purpose of dissolving the formazan crystals, and then the wells were placed in an incubator for 20 min. An ELISA plate reader was used at 500–600 nm for measurement of the absorbance. Wells with a higher absorbance depicted higher cell viability, while wells with low absorbance suggested low cell viability.

#### 2.8.2. Gene expression through real-time polymerase chain reaction 

After performing the MTT assay, the extract that exhibited the maximum cytotoxic effect, i.e. the ethanol extract (100 µg/mL), was further evaluated to check the expression of antiapoptotic [protein kinase B (Akt), B-cell lymphoma 2 (BCL2)] and proapoptotic [BCL2 associated X (BAX)] genes through real-time polymerase chain reaction (RT-PCR). The cells were plated with ethanol extract for 24 and 48 h, excluding the cells in the control well. Thereafter, the cells were harvested for ribonucleic acid (RNA) isolation after 24 and 48 h of exposure. As evident in the results, the morphology of the HepG2 cells, which had been incubated with ethanol extract, changed after 24 and 48 h when compared to the control, which contained healthy cancer cells.

#### 2.8.3. RNA extraction with trizol-reagent

RNA was extracted using trizol-reagent according to the manufacturer’s instructions. The cells were lysed in the wells by adding 1 mL of trizol reagent and collected in Eppendorf tubes. Later, the samples were homogenized and incubated at room temperature for 5 min. Into the Eppendorf tubes, 200 µL of chloroform was added, and then the tubes were shaken by hand for 15 s and incubated at room temperature for 2–3 min, and later centrifuged at 12,000 *g* for 15 min at 4 °C. The upper aqueous phase was transferred into a fresh Eppendorf tube and RNA was precipitated by adding 500 µL of isopropanol into each tube. The tubes were centrifuged at 12,000 *g* for 10 min at 4 °C and then later incubated at room temperature for 10–20 min. The supernatant was removed and the pellets were washed by adding 1 mL of ethanol (70%). They were mixed by vortexing and then centrifuged at 7500 *g* for 5 min at 4 °C. The supernatant was decanted and the tubes were dried, and the RNA was suspended in 20–30 µL of purified, RNAase-free water.

#### 2.8.4. Complementary DNA

The RNA was quantified and cDNA was prepared using the HyperScript first strand synthesis kit (Thermo Fisher Scientific Inc., Waltham, MA, USA; catalogue number 601-005), and reaction was conducted for 500 ng of RNA. Next, 1 µL of Oligo dT (50 µM), 1 µL of random hexamer (50 ng/µL), and 1 µL of dNTPs (10 mM) were added to the RNA (calculated based on 500 ng) in a PCR tube. The volume of the reaction mixture was adjusted to 14 µL with PCR water. The mixture was incubated at 65 °C for 5 min and allowed to cool on ice for 1 min. After that, 2 µL of RTase reaction buffer (10X), 2 µL of DTT (0.1M), 1 µL of reverse transcriptase (200 U/µL), and 1 µL of RNase inhibitor were added and reaction was conducted at 40–60 °C for 30–60 min. The reaction was terminated via incubation at 85 °C for 5 min, with subsequent chilling on ice.

#### 2.8.5. Real-time polymerase chain reaction

RT-PCR was performed as described by Saeed et al. [38], using specific primers for Akt, BCL-2, BAX, and beta-actin. The forward primer sequence for Akt was 5’‑TCT ATG GCG CTG AGA TTG TG‑3’, reverse primer sequence was 5’‑CTT AAT GTG CCC GTC CTT GT‑3’,BCL2 forward primer sequence was (5’ sense): 5’-CCT GTG GAT GAC TGA GTA CC-3’, BCL2 reverse primer sequence (3’ antisense): 5’-GAG ACA GCC AGG AGA AAT CA-3’, BAX forward primer sequence was (5’ sense): 5’-GTT TCA TCC AGG ATC GAG CAG-3’, BAX reverse primer sequence (3’ antisense): 5’-CAT CTT CTT CCA GAT GGT GA-3’, beta-catin forward primer sequence was 5’-AGA GCT ACG AGC TGC C TGAC-3’, and reverse sequence was 5’-AGC ACT GTG TTG GCG TAC AG-3’.

Quantitative RT-PCR for the expression of the genes was performed as described previously (Saeed et al., 2012). RT-PCR was performed on a MyiCycler thermal cycler (Bio-Rad, Hercules, CA, USA) using iQTM SYBR Green supermix (Bio-Rad), according to the manufacturer’s instructions. Quantification of the target and reference genes (β-actin) was performed in duplicate. Following normalization to the β-actin gene, the expression levels for each target gene were calculated using the comparative threshold cycle (CT) method [(1/ (2Δ CT), where ΔCT is the difference between the CT target and the CT (Saeed et al., 2011). Data were analyzed using Optical System 3.1 (Bio-Rad) software.

#### 2.8.6. Statistical analysis

All analyses were performed at least in technical triplicate, to estimate the mean and standard derivation (±SD). Microsoft Excel 2010 (Microsoft Corp., Redmond, WA, USA) was used to determine the mean value, standard deviation, and probability value. The means of two groups were compared using the Student paired t-test. For the median inhibitory concentration (IC_50_) measurement of the enzyme inhibitory activities, the nonlinear regression method was used, employing GraphPad prism (San Diego, CA, USA). α ≤ 0.05 was considered statistically significant.

## 3. Results and discussion

### 3.1. Physicochemical analysis

The powdered bark of *Olea ferruginea* R was evaluated physiochemically and the results revealed 7.58% moisture, 12% total ash, and 14.13% sulphated ash, and the alcohol soluble extractive value was higher than the water soluble extractive value (Table 1).

**Table 1:** Physicochemical properties and mineral contents of powdered bark of Olea ferruginea R.

Physicochemical properties							
Sample powder	Moisture content(% content ± SD)	Total ash content(% content ± SD)	Water soluble ash(% content ± SD)	Acid insoluble ash(% content ± SD)	Sulphated ash(% content ± SD)	Alcohol soluble extractive value(% content ± SD)	Water soluble extractive value(% content ± SD)
Olea ferruginea R	7.58 ± 0.10	12.02 ± 0.17	11 ± 0.2	4.59 ± 0.36	14.13 ± 0.32	7.1 ± 0.11	2 ± 0.06
Mineral content (ppm)							
	Calcium	Magnesium	Potassium	Iron	Copper	Lead	Zinc
Olea ferruginea R	18.31	9.63	8.94	0.22	0.08	0.03	–0.20 (ND)

### 3.2. Phytochemical studies

The powdered bark of *Olea ferruginea* R underwent the process of hot and cold extraction in order to obtain all of the thermo stable and thermo labile compounds. The phytochemical studies demonstrated that among the primary metabolites, carbohydrates were present in higher concentrations when compared to the proteins and lipids (Table 2). Evaluation of the secondary metabolites revealed that the chloroform extract was enriched in polyphenols (35.61 mg/g ± 0.03) and flavonoids (64.33 mg/g ±0.35), and the ethanol extract contained the highest concentration of polysaccharides (268.75 mg/g ± 0.34), while the concentration of glycosaponins was found to be highest in the methanol extract (78.01 mg/g ± 0.07) (Table 2). These results depicted that the *Olea ferruginea* R bark extracts were a good source of primary and secondary metabolites. As secondary metabolites have a major contribution in exhibiting therapeutic effects [39], they are expected to show desirable pharmacological activities, such as antidiabetic and anticancer.

**Table 2:** Estimation of primary and secondary metabolites of powdered bark of Olea ferruginea R.

Primary metabolites				
Sample powder	Total proteins(% content ± SD)	Total lipids(% content ± SD)	Total carbohydrates(% content ± SD)	
Olea ferruginea R.	36.01 ± 0.10	0.82 ± 0.08	43.68 ± 0.18	
Secondary metabolites				
Sampleextracts	Total polyphenols (mg/g ± SD)	Total flavonoids(mg/g ± SD)	Total polysaccharides (mg/g ± SD)	Total glycosaponins (mg/g ± SD)
n-Hexane	28.49 ± 0.04	38.09 ± 0.11	33.06 ± 0.07	1.06 ± 0.11
Chloroform	35.61 ± 0.03	64.33 ± 0.35	156.235 ± 0.25	74.06 ± 0.4
Methanol	28.33 ± 0.18	14.71 ± 0.29	195.66 ± 0.31	78.01 ± 0.07
Ethanol	26.15 ± 0.05	11.13 ± 0.16	268.75 ± 0.34	76.93 ± 0.4
Water	27.04 ± 0.06	8.11 ± 0.13	30.25 ± 0.25	72.02 ± 0.2

### 3.3. In vitro antidiabetic and anticancer activity

Medicinal plants serve as a continuous source of both traditional and modern-day medicine. Despite therapeutic headway, both diabetes and cancer are the leading causes of morbidity and mortality worldwide due to suboptimal treatment-related outcomes [40,41], and serious chemotherapy- and radiation therapy-related hazardous effects on normal cells [u1d96]. It is assumed that plant-based treatments for diabetes and cancer would confer minimal treatment-related side effects, and hence, improved compliance [u1d97]. This study has focused on the antidiabetic and cytotoxic activities of *Olea ferruginea* R bark extracts. 

#### 3.3.1. Nonenzymatic glycosylation of hemoglobin assay

**Figure 1:**
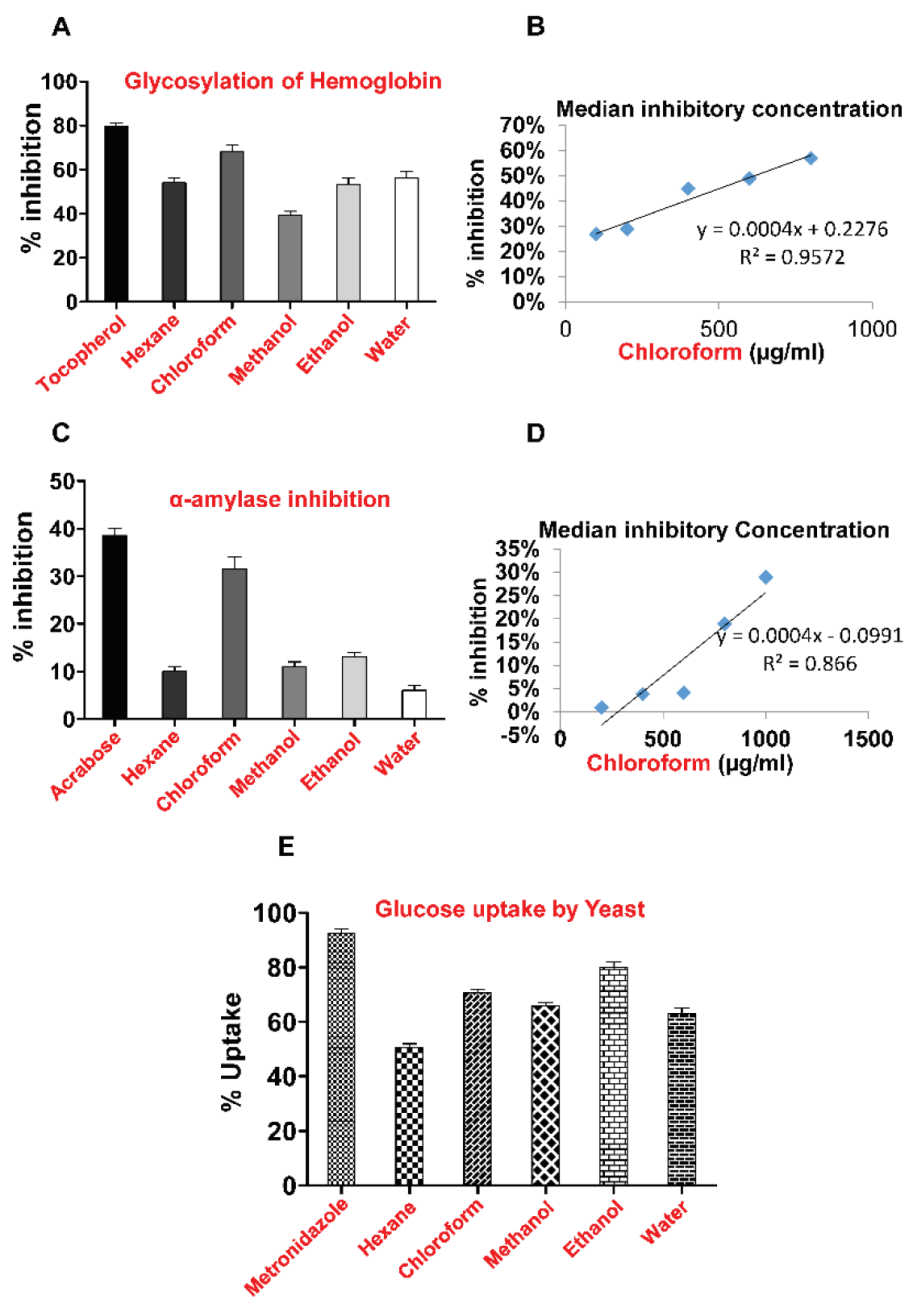
Antidiabetic effects of Olea ferruginea R bark extracts. A) Percentage (%) inhibition via nonenzymatic glycosylation of hemoglobin assay. B) Dose response relationship of the chloroform extract via nonenzymatic glycosylation of hemoglobin assay. C) Percentage (%) inhibition of the alpha-amylase enzyme. D) Dose response relationship of the chloroform extract via alpha-amylase enzyme assay. E) Percentage (%) inhibition of the glucose uptake by all of the extracts.

Nonenzymatic glycosylation involves the production of free radicals leading towards increased oxidative stress, which is the main contributing factor in developing diabetes [46]; hence, its inhibition is mandatory in order to prevent the ongoing disease condition. The nonenzymatic glycosylation of hemoglobin assay was performed on all 5 *Olea ferruginea* R bark extracts. The highest percentage of inhibition of nonenzymatic glycosylation of the bark extracts was observed for chloroform (65%), followed by hexane (56%), water (53%), ethanol (50%) and methanol (38%) (Figure 1A). Thus, the chloroform extract was the most active extract, with results comparable to the standard drug, i.e. tocopherol (Figure 1A). It was further evaluated for its dose response relationship, which suggested that with an increase in the concentration, its percentage inhibition increased, while the IC_50_ was achieved at 681 µg/mL (Figure 1B). Polyphenols are key compounds that lower the process of glycosylation [47]. Flavonoids obtained from natural products scavenge free radicals to confer antioxidant properties that might hinder the process of glycation [48]. As chloroform was the most active among the extracts, presumably, large amounts of polyphenols and flavonoids were present in it, as evident after the phytochemistry analysis, which may have been the reason behind its potent glycosylation inhibition.

#### 3.3.2. Alpha-amylase inhibition assay

The antidiabetic potential of *Olea ferruginea* R bark extracts were further assessed by alpha-amylase inhibition assay. The alpha-amylase enzyme assists in carbohydrate digestion [25]. According to a study conducted on soya bean extracts, polyphenolic compounds showed the outstanding potential of inhibiting the alpha-amylase enzyme [49]. The results of the in vitro alpha-amylase inhibition assay are shown in Figure 1C, according to which, the order of activity of all 5 extracts was found to be: chloroform (32%) > ethanol (12%) > hexane (11%) > methanol (10%) > water (7%). Acarbose was used as a standard, which showed the highest enzyme inhibition (40%) when compared to the plant extracts. Among the extracts, the chloroform extract inhibited the alpha-amylase enzyme more efficiently. The dose response relationship of the chloroform extract was determined, resulting in a IC_50_ of 1497 µg/mL (Figure 1D). Polyphenols are plant-derived hypoglycemic agents that block the action of alpha-amylase and alpha-glucosidase enzymes, thus preventing postprandial hyperglycemia [50]. Phytochemical screening of the chloroform extract revealed the presence of high concentrations of polyphenols, which can be among the contributing factors in attaining maximum enzyme inhibition. 

#### 3.3.3. Glucose uptake by yeast cells

In vitro antidiabetic assay, i.e. glucose uptake by yeast, was performed on all of the *Olea ferruginea* R bark extracts. The results of this assay revealed that among the 5 extracts, the ethanol extract showed the maximum increase in the percentage of glucose uptake, although it was less than the standard, i.e. metronidazole (Figure 1E). Plant-derived compounds, i.e. polyphenols, reveal their antidiabetic effects through the transport of glucose to the cells [51], while polysaccharides prolong the food digestion process, thereby decreasing glucose absorption [52]. As the ethanol extract possessed the maximum polysaccharide content, it might slow down the glucose absorption in the blood, consequently lowering serum glucose levels. 

#### 3.3.4. MTT assay

**Figure 2:**
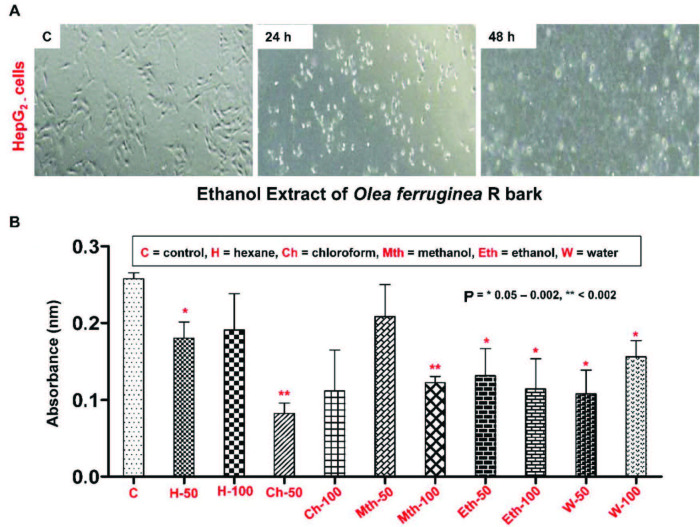
Cell survival and cytotoxicity assay of the HepG2 cells. A) Morphology of the control, and 24- and 48-h ethanolic extracttreated HepG2 cells showing the most significant effects. B) MTT assay of the HepG2 cells after treatment with different Olea ferruginea R bark extracts made in different solvents at 2 different concentrations, comprising 50 and 100 μg/mL.

Using the SRB protein stain assay, *Olea ferruginea* R leaf extracts were shown to possess anticancer activity against HeLa and vero cells, comparable to that of vincristine [16]. Herein, the cytotoxic potential of the *Olea ferruginea* R bark extracts were examined for the first time on HepG2 cells using the MTT assay, which did not stain the lysed cells like the SRB protein stain assay. The MTT assay suggested that the ethanol extract had higher anticancer activity against the HepG2 cells when compared to other extracts. As shown in Figure 2A, all of the extracts showed significant cytotoxic potential in comparison with the control. However, the ethanol extract exhibited the maximum anticancer effect in a dose-dependent manner, i.e. from 50 µg/mL to 100 µg/mL against the HepG2 cells (Figure 2A). The results of the MTT assay were corroborated by the morphological assessment of the HepG2 cells, with and without the addition of the extracts. As evident in Figure 2A, the control cancer cells appeared healthy with no apoptosis, while the cells in the ethanol extract appeared to be stressed, more flattened, with an increased number of dead cells with increased exposure time, i.e. 24–48 h. The MTT assay results clearly demonstrated that when compared to the control, the extract concentration of 50 µg/mL for hexane, chloroform, methanol, ethanol, and water had significantly reduced the absorbance, which was reflected by compromised cell viability due to the cytotoxic potential of these extracts (Figure 2B). However, only the ethanol extract demonstrated significant cytotoxic effects at both concentrations, i.e. 50 and 100 µg/mL (Figure 2B). Thus, the ethanolic extract (100 µg/mL) was further tested for the expression of genes with known involvement in cancer, Akt, BCL2, and BAX regulators of cell survival and death [53]. 

#### 3.3.5. Effect of Olea ferruginea R on Akt downstream proapoptotic and preapoptotic signals: 

**Figure 3:**
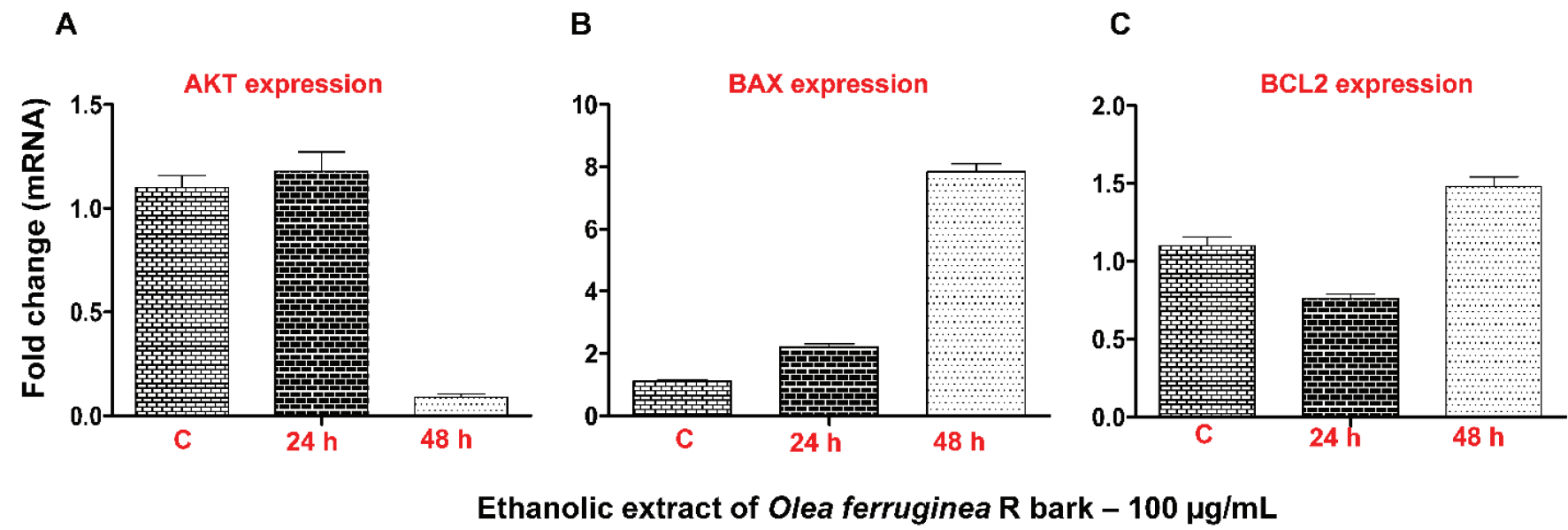
Expression of cell survival and apoptotic genes. A) Akt expression in the control and after 24- and 48-h exposure to the HepG2 cells. B) BAX expression in the control and after 24- and 48-h exposure to the HepG2 cells. C) BCL2 expression in the control and after 24- and 48-h exposure to the HepG2 cells.

The ethanol extract of *Olea ferruginea* R at a concentration of 100 µg/mL showed a significant cytotoxic effect in the HepG2 cells. Thus, the ethanol extract (100 µg/mL) was selected for expression analysis of prosurvival and proapoptotic genes with known involvement in cancer, such as Akt, BCL2, and BAX, after exposure to the HepG2 cells for 24 and 48 h. Gene expression was performed by RT-PCR using beta actin as a reference gene. The data from this study demonstrated that the Akt expression was higher after 24 h when compared to the control, but further exposure, until 48 h, resulted in a marked reduction in Akt expression (Figure 3A). On the other hand, the BAX expression demonstrated a continuous increase from 24 to 48 h in comparison with the control (Figure 3B). While, the BCL2 expression was reduced in 24 h, but later showed a marked increase after 48 h (Figure 3C).

As per the literature evidence, polysaccharides obtained from natural products not only inhibit tumor formation and initiate apoptosis in cancer cells, but also cease the process of metastasis within them [54]. Moreover, polysaccharides increase immunity in the body and impart a positive impact on human health along with the upregulation of tumor suppressor genes [55]. The ethanol extract was enriched in polysaccharides; hence, it is highly likely that the dose-dependent cytotoxic effects were due to these polysaccharides. Akt has been shown to inhibit apoptosis in tumor cells by triggering murine double minute-2, which in turn degrades apoptosis-causing protein p53, transcribing the nuclear factor κB gene, which is an apoptosis inhibitor, and reestablishing the activity of BCL-XL (apoptosis suppressor) via the inhibition of the proapoptotic gene (BAD) [56]. Data from the present study demonstrated that the Akt expression was higher after 24 h exposure when compared to the control, which may have been due to the inactivity of the phosphatase and tensin homolog (PTEN) protein in the HepG2 cells, which basically downregulates Akt function, but further exposure, until 48 h, resulted in a marked reduction in the Akt expression, either because of the presence of sufficient PTEN protein or the direct effect of the ethanolic extract on the Akt gene [57]. BCL2 displayed mixed expression pattern, downregulation after 24 h followed by upregulation after 48 h in comparison to control. It is highly likely that the downregulation of Akt might have resulted in the upregulation of BCL2 after 48 h exposure, since BCL2 lies down stream of Akt in an attempt to counter proapoptotic signals. As per the literature reports, p53, a tumor suppressor gene, decreased the expression of BCL2, probably due to modulation in the p53 function [58]. Moreover, it is likely that the HepG2 cells, like many cancerous cell lines, tended to resist the proapoptotic signals of the ethanolic extract. Moreover, Akt has been shown to regulate the expression of BAX, and herein, it was observed that the BAX expression decreased gradually from 24 to 48 h in comparison with the control. The increased expression of BAX after 48 h may have been due to the reduction in Akt expression or corelated to its phosphorylation, as the result of c-Jun N-terminal kinases and p38 protein kinase ultimately triggering its function and initiating apoptosis in the cancer cells [59]. Moreover, it cannot be ruled out that tumor suppressor gene p53 may have caused transcriptional upregulation of BAX after 48 h, which may have been attributed to decreased expression of Akt and the upregulation of p53 after 48 h exposure [60]. This study provided baseline data regarding the probable mechanism of the cytotoxic effects of the ethanolic extract of *Olea ferruginea* R bark, which will be helpful in conducting further in vivo studies to unravel the underlying mechanisms of its antidiabetic and anticancer effects. 

## 4. Conclusion

In the present study, an investigation of the physicochemical, phytochemical, in vitro antidiabetic, and anticancer activity was conducted on the bark of *Olea ferruginea* R. The phytochemistry of the powdered bark revealed that the primary metabolites were enriched in total carbohydrates, while the secondary metabolites were enriched in total polyphenols and total flavonoids. The highest concentration of total polysaccharides was present in the ethanol extract, while the methanol extract contained a huge amount of glycosaponins. The in vitro antidiabetic and anticancer activity data showed that the *Olea ferruginea* R bark extracts in chloroform and ethanol were comparable to the standard drugs with regards to the nonenzymatic glycosylation of hemoglobin assay, alpha-amylase inhibition assay, and glucose uptake by yeast cells. Similarly, when compared to the other extracts, the ethanol extract was sufficiently efficient to show cytotoxic potential against the HepG2 cells, which was attributable to the lower and higher expression of Akt and BAX, respectively. This study provided a baseline data to conduct more detailed analysis of the extracted constituents followed by in vivo antidiabetic and anticancer investigations. 
